# Effect of 4-week preoperative prism adaptation in preventing postoperative residual esotropia

**DOI:** 10.1186/s12886-024-03490-x

**Published:** 2024-05-27

**Authors:** Bosook Han, Joo Yeon Lee

**Affiliations:** Department of Ophthalmology, Hallym University College of Medicine, Hallym University Sacred Heart Hospital, 22, Gwanpyeong-ro 170 beon-gil, Dongan-gu, Anyang, Republic of Korea

**Keywords:** Esotropia, Surgery, Target angle, Prism adaptation

## Abstract

**Background:**

Preoperative prism adaptation (PPA) simulates postoperative status and possibly can predict postoperative undercorrection before surgery in esotropia. The present study aimed to assess the effect of 4-week PPA in preventing postoperative residual esotropia.

**Methods:**

Seventy-five (75) esotropes who had undergone surgery at a single strabismus center were retrospectively enrolled. They included 25 basic, 31 acute comitant, 10 partially accommodative, and 9 recurrent esotropia patients. The preoperative deviation angle, which had been determined using the alternating prism and cover test, was fully corrected with press-on prisms 4 weeks before surgery. If there was an increase of 5 PD or more of esodeviation, the prisms were changed accordingly at 2 weeks. The deviation angle measured at 4 weeks was determined as the surgical target angle. Patients were then divided into increase (≥ 5 PD increase of angle during 4-week PPA) and non-increase groups. Success was defined as either esodeviation of 8 PD or under or exodeviation of 5 PD or under at distance at postoperative 6 months.

**Results:**

The increase group included 44 patients (58.7%). The mean deviation angle before PPA was 27.4 PD, and after the 4-week PPA, there was an average increase of 9.4 PD. The success rate was 90.9% in the increase group and 96.8% in the non-increase group (*p* = 0.316). There were no intergroup differences in preoperative clinical characteristics, esotropia types, postoperative deviation angle or postoperative near stereopsis (*p* > 0.05).

**Conclusions:**

The results of this study indicated a beneficial effect of 4-week PPA in esotropia of various types, specifically by uncovering the hidden esodeviation in the increase group and simulating the postoperative alignment in both the increase and the non-increase groups.

## Background

Prisms are used to treat acquired esotropia by improving binocular vision and reducing diplopia symptoms. Also, the use of prisms before surgery has been suggested as a means of improving surgical outcomes, specifically by providing an estimation of fusional potential and an accurate target angle.

Esotropia patients need to adapt to the anomalous horizontal input between the two eyes and learn to adjust the motor fusion to compensate for that disparity. Sometimes, accurate and careful examination by an expert cannot reveal the total deviation angle, because the adaptation mechanism could not be sufficiently broken. Thus, unexpected occurrence of early-postoperative undercorrection would be a dilemma in esotropia surgery. Augmented surgeries and prism adaptation test (PAT) have been chosen as the major options to reduce undercorrection rate [1–4].

Although the PAT test before esotropia surgery has been reported to have benefits in improving surgical outcomes, there remain questions concerning preoperative prism adaptation (PPA) in such surgery. Are the beneficial effects on surgical outcomes attributable to reinforced binocular fusion or to increased surgical amount with the achieved built-up angle, or both? What is the most effective prism adaptation period? Are there any specific types of patients for whom the PAT is efficacious for different types of esotropia or certain clinical variables?

We applied 4-week PPA in order to help break the adapted motor fusion and, thereby, uncover latent deviation if any existed. The 4-week PPA also was applied to prevent postoperative residual esotropia by simulating early-postoperative status after surgery and predicting the postoperative undercorrection. We performed the 4-week PPA in concomitant acquired esotropia of various types in order to obtain a surgical target angle. This study aimed to assess surgical results for various types of esotropia based on the maximum prism-adapted angle after 4-week PPA.

## Methods

We retrospectively reviewed the medical records of patients who had been diagnosed with esotropia and subsequently underwent surgery at a single strabismus center of a tertiary referral hospital between January 2014 and January 2022. The major inclusion criteria were (i) patients who had undergone surgery for treatment of esotropia, (ii) patients with concomitant esotropia including basic esotropia, partially accommodative esotropia, acute acquired concomitant esotropia or recurrent esotropia after primary surgery for concomitant esotropia, (iii) surgery based on the target angle of esotropia after 4-week PPA, and (iv) at least 6 months of follow-up after surgery. Among concomitant esotropia, basic esotropia is defined as a form of acquired nonaccommodative esotropia with a gradual onset that does not manifest as an acute symptom with diplopia. It is differentiated from acute acquired concomitant esotropia, which is accompanied by an acute onset with diplopia within 6 months.

The exclusion criteria were (i) congenital(infantile) esotropia, (ii) incomitant esotropia such as paralytic or restrictive strabismus and A- or V-pattern strabismus, (iii) esotropia with high AC/A ratio showing a distance-near difference of more than 10 prism diopters (PD), (iv) untreated amblyopia with best-corrected vision in any eye worse than 20/25, and (v) previous surgical history for strabismus except primary surgery for acquired concomitant esotropia.

This retrospective case series conformed to the Declaration of Helsinki, and was approved by the Institutional Review Board (IRB) of Hallym University Sacred Heart Hospital, informed consent having been waived (IRB No. 2023-04-016). The clinical data were collected from medical records, and included sex, age, esotropia type, surgery type, preoperative refractive error, near stereopsis, and angle of deviation. Near stereopsis (arc/sec) was measured using the Titmus-fly test.

Prior to the surgery, PPA was performed over the course of 4 weeks as follows. First, we confirmed the largest angle of esotropia measured by alternating prism and cover test (APCT) during at least three consecutive follow-up visits that occurred at intervals of a couple of months. The APCT was performed with the patient wearing their own glasses for best refractive correction and with an accommodative target of 20/40-size Snellen letters at a distance of 6 m. Once the surgery schedule was determined, press-on prisms(3 M Company St Paul, MN55144 USA) according to the largest angle of distance esodeviation were mounted on the patients’ glasses for the 4 weeks prior to the surgery. Patients who did not need to wear corrective glasses wore plano glasses mounted with press-on prisms. The press-on prisms were placed on the fixing eye if it was 30PD or under. If the power was over 30 PD, the prisms were divided equally between the two eyes, or the larger one was placed on the fixing eye with the lesser one on the deviating eye. The deviation angle at this time was designated as the baseline angle.

The APCT was repeated 2 weeks after prism wear, and if the angle had increased by 5 PD or more from the baseline angle, the press-on prisms were changed accordingly and the patents were followed-up at 4 weeks. The deviation angle, which was measured by APCT while wearing prism-mounted glasses plus the prism power of mounted press-on prisms at 4 weeks after prism wear, was defined as the surgical target angle.

We classified patients into increase and non-increase groups according to their response to the 4-week PPA: the increase group, based on an esodeviation angle increase of 5 PD or more from the baseline angle at 2 weeks after prism wear, or 4 weeks, or both; or the non-increase group, based on a change of esodeviation angle of less than 5 PD from the baseline angle at both 2 and 4 weeks.

A single strabismus surgeon performed all of the procedures for measurement of esodeviation angle as well as the surgeries under general anesthesia. Adjustment suture techniques were not used. The operation selected was bilateral medial rectus (BMR) recession, bilateral lateral rectus (BLR) resection, or 3-muscle surgery in the case of large deviation of more than 50 PD. The amount of muscle recession or resection followed guidelines suggested in the standard dose-response table (Table 1) [5]. Patients were routinely followed-up at 1 day, 2 weeks, 1, 3 and 6 months after surgery, and then every 6 months after that.

**Table 1:** Surgical dosages for acquired concomitant esotropia

Esodeviation (PD)	BMR recession (mm)	BLR resection (mm)
17–20	3.5	4.5
21–25	4	5.5
26–30	4.5	6
31–35	5	6.5
36–40	5.5	-
41–45	6	-
46–50	6.5	-

*BMR = bilateral medial rectus (muscles)

*BLR = bilateral lateral rectus (muscles)

A successful surgical result was defined as orthotropia, or an esodeviation angle of 8 PD or under, or an exodeviation angle of 5 PD or under at distance. Surgical success was defined as a successful result at postoperative 6 months. The primary outcomes of this study undertaken to assess the effect of 4-week PPA were (1) the proportion of the increase group and (2) the surgical success rates in the increase and non-increase groups. The secondary outcomes included the comparisons of the surgical results between the increase and non-increase groups at postoperative 1 month, 6 months, and 1 year. Also compared between the two groups were potential clinical factors affecting PPA, including age, esotropia type, deviation angle, stereoacuity, and surgery type.

For the statistical analyses, SPSS version 27.0 was employed. Esotropic deviation was considered as a positive (+) variable, while exotropic deviation was considered as a negative (-) variable in statistical analysis. We compared the groups using the unpaired Student’s t-test for quantitative variables and the chi-square test for categorical variables. A paired Student’s t-test was performed to analyze the changes before and after surgery as well as the changes after prism adaptation. The results were considered statistically significant at *P*-values less than 0.05.

## Results

A total of 75 patients with esotropia were included in this study. Their demographics and characteristics are shown in Table 2. Forty-two (42) of 75 (56.0%) were male. The mean age at surgery was 22.07 ± 16.75 years. The mean baseline angle of esodeviation before PPA was 27.47 ± 10.38 PD at distance. Forty-four (44) patients (58.7%) comprised the increase group. Specifically, 21 patients (28.0%) showed an increase of esodeviation angle of 5 PD or more from the baseline angle at 2 weeks after prism wear, 9 patients (12.0%) at 4 weeks and 18 patients (18.7%) at both 2 and 4 weeks. Thus, the surgical target angle measured at 4 weeks after prism wear had been increased to 33.28 ± 11.74 PD from the baseline angle of 27.47 ± 10.38 PD.

The overall surgical success rate was 93.33% (70/75) at postoperative 6 months, 90.91% (40/44) for the increase group and 96.77% (30/31) for the non-increase group. There was no significant difference in surgical success between the increase and non-increase groups (*p* = 0.316) (Table 3). This study included 24 children(12 years old or under) and 51 adolescents/adults (13 years old and older). There were no statistically significant differences between the two groups regarding the percentage of the increase group versus the non-increase group (*p* = 0.968)(Table 3), nor in the success rate at the postoperative 6 months (*p* = 0.552). The success rate in children was 95.8% (23/24) and in adolescents/adults was 92.2% (47/51). The success rates for the four different types of esotropia were as follows: 96% for basic esotropia, 93.5% for acute acquired concomitant esotropia, 80% for partially accommodative esotropia, and 100% for recurrent esotropia (*p* = 0.285).

**Table 2:** Demographics and clinical characteristics of patients who had esotropia surgery with 4-week PPA*

Variables	*N* = 75
Sex (Male: Female)	42: 33 (56%: 44%)
Age at surgery (years)	22.07 ± 16.75 (range: 4 to 77)
Preoperative refractive error	
myopia (n, %)	45 (60.00%)
emmetropia (n, %)	7 (9.33%)
hypermetropia (n, %)	23 (30.67%)
Type of esotropia (n, %)	
Basic esotropia	25 (33.33%)
Acute comitant esotropia	31 (41.33%)
Partially accommodative esotropia	10 (13.33%)
Recurrent esotropia	9 (12.00%)
Preoperative initial angle before PPA (PD)	27.47 ± 10.38 (range: 15 to 55)
Final target angle of esodeviation for surgery (PD)	33.28 ± 11.74 (range: 16 to 62)
Positive response rate (%)	44 (58.7%)
At 2 weeks after prism wear	21 (28.00%)
At 4 weeks after prism wear	9 (12.00%)
At 2 & 4 weeks after prism wear	14 (18.67%)
Preoperative stereoacuity by Titmus test	309.86 ± 253.63 (range: 40 to 800)
Type of surgery (n, %)	
BMR recession	57 (76.00%)
BLR resection	8 (10.67%)
3-muscle surgery	10 (13.33%)

*BMR = bilateral medial rectus (muscles)

*BLR = bilateral lateral rectus (muscles)

Data presented as mean ± SD

The two groups’ clinical variables are shown in Table 3. As can be seen, the preoperative clinical characteristics were not significantly different between the groups (*p* > 0.05). For the increase group, after 4-week PPA, the mean esodeviation angle change was 9.39 ± 4.92 PD. So, the surgical target angle of esodeviation was significantly larger in the increase group (36.75 ± 10.97 PD) than in the non-increase group (28.35 ± 0.74 PD) (*p* = 0.002). The mean angle measured at 2 weeks after wearing prisms increased significantly to 30.64 ± 11.76 PD from the baseline angle of 27.47 ± 10.38 PD (*p* = 0.000). Furthermore, the surgical target angle measured at 4 weeks after prism wear showed a significant increase to 33.28 ± 11.74 PD compared to both the angle at 2 weeks (*p* = 0.00) and the baseline angle (*p* = 0.000). Neither the surgical success rate nor the mean postoperative angle of esodeviation showed any significant intergroup difference (*p* = 0.316 and *p* = 0.776, respectively). The patients who showed a positive response at 4 weeks and at both 2 and 4 weeks totaled 23 patients. Their surgical success rate was 91.3%(21/23), which was not statistically different from either the success rate of 90.5%(19/21) in the patients who showed positive response at 2 weeks (*p* = 0.924), or 96.8%(30/31) in non-increase group (*p* = 0.386). Postoperative stereoacuity as measured at postoperative 6 months was not statistically different between the groups (*p* = 0.794).

Among the five cases with surgical failure, four were due to undercorrection, with a mean deviation angle at postoperative 6 months of + 11.7 ± 2.36PD at postoperative 6 months. One case exhibited overcorrection of -12PD. All undercorrections occurred in the increase group and one overcorrection among the failures was observed in the non-increase group.

**Table 3:** Comparison of clinical variables between increase and non-increase groups

Variables	Increase group (*N* = 44)	Non-increase group (*N* = 31)	*P*-value
Sex (Male: Female)	27: 17	16: 15	0.400
Age at surgery (years)	20.38 ± 14.79	24.45 ± 19.21	0.304
Children (12 years old or under)	14	10	0.968
Adolescents/adults (13 years old or older)	30	21	
Preoperative refractive error			0.768
myopia (n, %)	26 (59.09%)	19 (61.29%)	
emmetropia (n, %)	5 (11.36%)	2 (6.45%)	
hypermetropia (n, %)	13 (29.54%)	10 (32.29%)	
Type of esotropia (n, %)			0.262
Basic esotropia	17 (38.64%)	8 (25.80%)	
Acute comitant esotropia	17 (38.64%)	14 (45.16%)	
Partially accommodative esotropia	7 (15.91%)	3 (9.68%)	
Recurrent esotropia	3 (6.82%)	6 (19.35%)	
Preoperative initial angle before PPA (PD)	27.36 ± 9.79	27.61 ± 11.31	0.919
Final target angle of esodeviation for surgery (PD)	36.75 ± 10.97	28.35 ± 0.74	0.002
Amount of angle change after 4-week PPA	9.39 ± 4.92	0.74 ± 1.37	
Preoperative stereoacuity by Titmus test	317.27 ± 250.30	298.62 ± 262.66	0.655
Type of surgery (n, %)			0.186
BMR recession	33 (75.00%)	24 (77.42%)	
BLR resection	3 (6.81%)	5 (16.13%)	
3-muscle surgery	8 (18.18%)	2 (6.45%)	
Postoperative angle of esodeviation, 6 months (PD)	2.45 ± 4.08	2.23 ± 2.85	0.776
Success rate at postoperative 6 months (%)	90.9%	96.8%	0.316
Postoperative stereoacuity by Titmus test	172.04 ± 161.67	157.42 ± 157.35	0.794

*BMR = bilateral medial rectus (muscles)

*BLR = bilateral lateral rectus (muscles)

Data presented as mean ± SD

At 1 year after surgery, follow-up loss was seen for 21 patients; the proportion of patients with successful results was 90.74% (49/54). There was no statistically significant difference in surgical results at postoperative 1 month, 6 months, and 1 year between the increase and non-increase groups (Table 4). There were no statistically significant differences in success rates between the postoperative 6 months and 1 year neither in the increase group (*p* = 0.708) nor in the non-increase group (*p* = 0.189). Table 5 shows the residual angle of esodeviation after surgery for each group. The mean postoperative angle was 2.45 ± 4.08 PD for the increase group and 2.22 ± 2.85 PD for the non-increase group at postoperative 6 months. There were no statistically significant intergroup differences of residual esotropia at 1 month, 6 months or 1 year after surgery (Table 5).

**Table 4:** Comparison of proportions of patients with successful results during 1-year postoperative follow-up between increase and non-increase groups

*n*/*N* (%)	Total	Increase group	Non-increase group	*P*-value
Postoperative 1 month	70/75 (93.33%)	41/44 (93.18%)	29/31 (93.55%)	0.950
Postoperative 6 months	70/75 (93.33%)	40/44 (90.91%)	30/31 (96.77%)	0.316
Postoperative 1 year	49/54 (90.74%)	28/30 (93.33%)	21/24 (87.50%)	0.462

**Table 5:** Comparison of postoperative angle (PD) at distance after surgery between increase and non-increase groups

	Increase group	Non-increase group	*P*-value
Postoperative 1 month	2.39 ± 3.64	2.13 ± 3.37	0.757
Postoperative 6 months	2.45 ± 4.08	2.23 ± 2.85	0.776
Postoperative 1 year	3.81 ± 4.58	3.50 ± 4.54	0.796

Data presented as mean ± SD (PD)

The sensory outcome was estimated by comparison of (1) preoperative near stereopsis as measured without press-on prisms before PPA with (2) postoperative near stereopsis. The mean stereopsis as measured by Titmus-fly test improved from 309.86 ± 253.63 arc/sec preoperatively to 166.27 ± 158.79 arc/sec after surgery (*p* < 0.001). Also, both the increase and non-increase groups showed significant improvement of mean stereopsis after surgery (*p* = 0.002, 0.032 respectively). With normal stereopsis defined as 60 s or less, 8 patients improved to normal stereopsis postoperatively from preoperative subnormal stereopsis. Nine patients showed normal stereopsis both before and after surgery. Also, there were no cases in which stereopsis worsened from the normal range before surgery to subnormal stereopsis after surgery. The postoperative near stereopsis was normal in 17 patients (22.7%), gross (200 s or better) in 44 patients(58.7%), and worse than 200 s in 14 patients (18.7%). The preoperative near stereopsis was normal in 9 patients (12.0%), gross (200 s or better) in 33 patients(44.0%), and worse than 200 s in 33 patients (44.0%). Among the 42 patients with normal or gross stereopsis, 27 were in increase group. Among the 33 patients with stereopsis worse than 200 s, 20 were in increase group (*p* = 0.744). Furthermore, among the 42 patients with normal or gross stereopsis, 40 showed surgical success. Among the 33 patients with stereopsis worse than 200 s, 30 showed surgical success (*p* = 0.649). These results indicate that preoperative near stereopsis was not correlated to response to PPA or surgical success.

## Discussion

With the 4-week PPA, 58.7% of patients showed a more than 5 PD-increased surgical target angle relative to the baseline angle of esodeviation, regardless of esotropia type, amount of baseline angle, preoperative stereopsis, age at surgery, or any other clinical characteristics. With targeting of the surgical amount by the result of the 4-week PPA, the surgical success rate was 93.33%. The increase and non-increase groups showed similar success rates, 90.91% for the former and 96.77% for the latter (*p* = 0.316). The overall surgical success rate for esotropia in this study, 93.33%, is comparable to the results reported elsewhere, which range from 65 ~ 100% [1, 4, 6–13]; it should be noted, however, that each study differed in number of eligible patients, length of follow-up and the definition of success.

The present study’s surgical success rate also is favorable when compared with previous studies specifically employing the PAT. Scott and Thalacker reported 82% surgical success with the deviation angle under 8 PD at 6 months after prism-adapted surgery in patients with acquired esotropia [1]. In the representative multicenter investigation by the Prism Adaptation Study (PAS) Research Group, a prism-adapted surgery referred to surgery for the angle of esotropia at which the patients achieved sensory fusion. Prism responders were defined as patients who had shown motor stability in a simultaneous prism and cover test as well as sensory fusion. The study showed a better success rate (0 to 8 PD esodeviation at 6 months after surgery) in prism responders (success rate 89%) than in prism non-responders (73%) or in patients for whom PPA had not been performed (72%) [9]. By the PAS Research Group, 90% of 1-year success rate among prism responders who had undergone surgery for the prism-adapted angle was recorded [6].

Much previous research has employed PATs based on the design of the PAS Research Group [6, 9]. In those studies, PATs were performed to estimate sensory-motor fusion and to define ‘responders’ as those who gain stable esophoria with retained sensory fusion by wearing prism glasses. The PPA test has been reported to have beneficial effects on surgical outcomes [1, 3, 6, 9]. However, the question of whether improved surgical outcomes are attributable to the fusional potential or to the increased surgical amount with the achieved built-up angle, or both, still does not have a clear answer. Also, when responders were considered to be patients who had sensory fusion at the adapted angle, the surgical amount in non-responders (without fusion) was based on the entry angle before PPA in some studies and on the built-up angle following PPA in some others. This diversity of treatment approaches for non-responders with no sensory fusion and built-up angle by PATs would tend to make the effects of PATs somewhat obscure.

Hwang et al. reported that 89% of prism responders and 100% of prism non-responders had successful ocular alignment after 1 year in cases of esotropia with hypermetropia [3]. They had defined prism non-responders according to the PAS Research Group’s definition; however, they did not define the surgical target angle as the entry angle before prism wear. Rather, the non-responders discontinued prism wear, and the esodeviation angle was measured before surgery. In contrast, the PAS Research Group reported an 89% success rate among prism responders who had undergone surgery for prism-adapted angle, and a 73% success rate among non-responders who had undergone surgery for entry angle as measured prior to prism wear [9]. Jang et al. [4] reported no post-PAT difference in 1-year surgical outcomes between patients who needed prism power add-up and those who did not need add-up in cases of partially accommodative esotropia (*p* = 0.299). They did not classify ‘non-responder’ according to sensory findings, and performed surgical corrections for distance deviation after PPA in all subjects. Similarly, in the present study, we did not classify subgroups according to sensory findings after the 4-week PPA, and we performed surgical corrections based on the maximum prism-adapted angle, regardless of sensory fusion with prism wear. And there was no significant difference in surgical success rates between the increase group and the non-increase group. In view of these findings, it seems that the beneficial effect of PPA in improving surgical results largely depends on the prism-adapted angle rather than on fusion. PATs could be a surgical enhancement option to determine the maximum target angle by simulating the occurrence of postoperative residual esotropia. We suppose that the surgical outcome of the prism non-responders in the PAS Research Group studies would have been improved if the surgery had been performed for the post-PAT angle instead of the entry angle. Velez FG and Rosenbaum AL reported 100% of success rate in acquired esotropia and Kassem RR reported 95% of success rate in acquired esotropia with surgery for the prism-adapted angle regardless whether the prism responder or non-responder after the prism adaptation test [10, 11]. Both studies emphasized that surgery for the prism adapted angle in non-responders should be beneficial.

The success rates in this study did not show significant differences between esotropia types (*p* = 0.285), ranging from as low as 80% partially accommodative esotropia to as high as 100% in recurrent esotropia. Previous studies have reported success rates for standard esotropia surgery without preoperative prism adaptation tests ranging from 65.7–92% [7, 12, 13] for nonaccommodative acquired esotropia, 49–70.9% [14–16] for partially accommodative esotropia, and 52–68% [17–20] for recurrent esotropia. Comparing the success rates after 4-week PPA in this study with previously reported success rates after standard surgery, it seems that the recurrent esotropia achieves relatively high benefit from surgery using the 4-week PPA protocol. However, the number of cases was too small, therefore, further investigation will be warranted in future studies.

We did not perform the simultaneous prism and cover test nor the Worth-4-Dot test to evaluate binocularity with press-on prism wear in the present study, because we did not account for the test results in determining the surgical amount by the 4-week PPA. We had considered that the 20/40 target size in the APCT in wearing of prism glasses was small enough to prevent erroneous angles by perifoveal fixation, and thus also, that the measured angle could be taken as an exact amount of esodeviation with foveal fixation in each eye.

This study applied 4-week PPA because we had estimated that this duration could simulate early-postoperative status after surgery and, thereby, predict postoperative undercorrection with unmasked latent deviation. In the increase group, the significant increases in deviation angle were observed from baseline to 2 weeks, from baseline to 4 weeks, and from 2 weeks to 4 weeks. These findings suggest that there is a significant increase in deviation angle over time, indicating the efficacy of prism wear. Additionally, the significant increase in angle measurements between 2 and 4 weeks suggests that a minimum duration of 4 weeks may be necessary for optimal results compared to a 2-week timeframe. The surgical success rate and mean postoperative angle of esodeviation showed no significant intergroup difference (*p* = 0.316 and *p* = 0.776, respectively). This meant that the lack of enhanced surgery amount in the non-increase group did not result in more residual esodeviation than in the increase group (with increased amount of surgery) following the 4-week PPA. We think these results mean that the 4-week duration was sufficient to confirm that there was no latent deviation in the non-increase group. In increase group, the results were somewhat complex. The increase group had a lower success rate at postoperative 6 months than did the non-increase group, though it was not statistically significant. If we had had a longer PPA time duration, the alignment instability may have been further revealed in some increase group cases. Despite this hypothesis, the observed surgical success rates did not exhibit a statistically significant difference between patients with a positive response at 4 weeks and those without. This finding suggests that the 4-week prism adaptation duration was sufficient to predict postoperative outcomes in our cohort. However, it is important to acknowledge that while statistical significance was not reached, there may still be clinical significance in the trend towards a lower success rate in the increase group. The lack of statistical significance may be attributed to the relatively small sample size. A prospective study involving 32 patients with acquired esotropia investigated whether the prism adaptation process could be condensed into a shorter timeframe, ranging from 24 h to 7 days than traditionally prescribed in the PAS. They observed the motor stability excluding sensory adaptation, and found that stable motor alignment could be achieved within 24 h for the majority of patients, and 2 patients (6%) required a prism change to adjust the angle on the 7-day visit [21]. Additionally, 12 patients remained stable without any deviation change through the 7-day period. It is conceivable that extending the prism adaptation duration could have provided further elucidated alignment instability within this subgroup. Nevertheless, the exploration of a shorter prism adaptation timeframe in the prospective study raises intriguing possibilities. The optimal duration of prism adaptation remains an area of ongoing exploration and debate in the field. There are still no standard guidelines for PPA time. PPA time ranges from 30 min to several weeks in previous reports, no consensus having been achieved [3, 4, 9, 21–25]. This issue should be examined in future studies.

Our study has some limitations. The main one is its nature as a retrospective single-center study. The relatively small sample size and bias of the retrospective data collection would have reduced the power to prove the statistical results. Also, this study could not establish a control group that did not undergo 4-week PPA, because most of the patients with acquired esotropia had already undergone it at the strabismus center. Therefore, further prospective multicenter studies with a larger sample size and appropriate control patients could be helpful for distinguishing more accurate performance and examining the effectiveness of PPA for esotropia patients.

## Conclusions

In conclusion, 4-week PPA for acquired esotropia of various types improved surgical results by uncovering the latent esodeviation in the increase group and simulating the postoperative alignment in both the increase and non-increase patient groups. More than half of the patients (58%) were in the increase group, and they showed no significant clinical distinctions from the non-increase group. Even if 4-week PPA takes some effort and time, it does enhance the accuracy of the determined surgical amount of recession.

## Data Availability

No datasets were generated or analysed during the current study.

## Abbreviations

APCT: Alternating prism and cover test
BMR: Bilateral medial rectus (muscles)
BLR: Bilateral lateral rectus (muscles)
D: Diopters
PAS: Prism Adaptation Study
PAT: Prism adaptation test
PD: Prism diopters
PPA: Preoperative prism adaptation
SD: Standard deviation
SE: Spherical equivalent

## References

[1] Scott WE, Thalaker JA (1984). Preoperative prism adaptation in acquired esotropia. Ophthalmologica.

[2] Wright KW, Bruce-Lyle L (1993). Augmented surgery for esotropia associated with high hypermetropia. J Pediatr Ophthalmol Strabismus.

[3] Hwang JM (2001). A randomized comparison of prism adaptation and augmented surgery in the surgical management of esotropia associated with hypermetropia: one- year surgical outcomes. J AAPOS.

[4] Jang YJ, Lee HJ, Jung JH, Kim SJ (2021). Effect of prism adaptation in patients with partially accommodative esotropia: clinical findings and surgical outcomes. Graefe’s Archive Clin Experimental Ophthalmol.

[5] Wright KW. Color atlas of ophthalmic surgery. Strabismus. Philadelphia (PA): Lippincott;1991. P.241.

[6] Repka MX, Connect JE, Scott WE, Prism Adaptation Study Research Group (1996). The one-year surgical outcome after prism adaptation for the management of acquired esotropia. Ophthalmology.

[7] Kim DH, Noh HJ (2021). Surgical outcomes of acute acquired comitant esotropia of adulthood. BMC Ophthalmol.

[8] Aslihan U, Asena KS (2021). Factors influencing surgical success in concomitant horizontal strabismus. Acta Med.

[9] Prism adaptation Study Research Group (1990). Efficacy of prism adaptation in the surgical management of acquired esotropia. Arch Ophthalmol.

[10] Kassem RR, Elhilali HM, El-Sada MA, El-Antably SA (2020). A comparative study of prism adaptation and the augmented surgery formula in the management of acquired comitant esotropia. J Pediatr Ophthalmol Strabismus.

[11] Velez FG, Rosenbaum AL (2002). Preoperative prism adaptation for acquired esotropia: long-term results. J AAPOS.

[12] Kim E, Choi DG (2017). Outcomes after the surgery for acquired nonaccommodative esotropia. BMC Ophthalmol.

[13] Sturm V, Menke MN, Knecht PB, Schoffler C (2011). Long-term follow-up of children with acute acquired concomitant esotropia. J AAPOS.

[14] Kurup SP, Barto HW, Myung G, Mets MB (2018). Stereoacuity outcomes following surgical correction of the nonaccommodative component in partially accommodative esotropia. J AAPOS.

[15] AlShammari S, Alaam M, Alfreihi S (2022). Conventional surgery versus botulinum toxin injections for partially accommodative esotropia. JAAPOS.

[16] Mohan K, Sharma SK (2018). Long-term motor and sensory outcomes after surgery for the nonaccommodative component of partially accommodative esotropia. JAAPOS.

[17] Biedner B, Yassur Y (1987). Effect of resection of lateral rectus muscle in undercorrected esotropia. Ophthalmologica.

[18] Gunasekera LS, Simon JW, Zobal-Ratner J, Lininger LL (2002). Bilateral lateral rectus resection for residual esotropia. JAAPOS.

[19] Jang GJ, Park MR, Park SC (2004). Bilateral lateral rectus resection in patients with residual esotropia. Korean J Ophthalmol.

[20] Kassem RR (2020). A pilot study of the value of prism adaptation in planning strabismus reoperations. J Pediatr Ophthalmol Strabismus.

[21] Altman M, Baker JD, Petrunak J, Schweers M (1999). Can prism adaptation for acquired esotropia be accomplished in a short time frame?. J AAPOS.

[22] Takada R (2021). Efficacies of preoperative prism adaptation test and monocular occlusion for detecting the maximum angle of deviation in intermittent exotropia. BMC Ophthalmol.

[23] Repka MX (2021). Predictors of prism response during prism adaptation. J Pediatr Ophthalmol Strabismus.

[24] Langenthal AS (1989). Preoperative prism adaptation test in normosensoric strabismus. Graefe’s Arch Clin Exp Ophthalmol.

[25] Ela-Dalman (2006). Maximum motor fusion combined with one-hour preoperative prism adaptation test in patients with acquired esotropia. J AAPOS.

